# Testing Theory of Planned Behavior and Neo-Socioanalytic Theory models of trait activity, industriousness, exercise social cognitions, exercise intentions, and physical activity in a representative U.S. sample

**DOI:** 10.3389/fpsyg.2015.01114

**Published:** 2015-08-06

**Authors:** Phuong T. Vo, Tim Bogg

**Affiliations:** Department of Psychology, Wayne State University, DetroitMI, USA

**Keywords:** personality, social cognition, Theory of Planned Behavior, Neo-Socioanalytic Theory, industriousness, trait activity, physical activity

## Abstract

Prior research identified assorted relations between trait and social cognition models of personality and engagement in physical activity. Using a representative U.S. sample (*N* = 957), the goal of the present study was to test two alternative structural models of the relationships among the extraversion-related facet of activity, the conscientiousness-related facet of industriousness, social cognitions from the Theory of Planned Behavior (perceived behavioral control, affective attitudes, subjective norms, intentions), Social Cognitive Theory (self-efficacy, outcome expectancies), and the Transtheoretical Model (behavioral processes of change), and engagement in physical activity. Path analyses with bootstrapping procedures were used to model direct and indirect effects of trait and social cognition constructs on physical activity through two distinct frameworks – the Theory of Planned Behavior and Neo-Socioanalytic Theory. While both models showed good internal fit, comparative model information criteria showed the Theory-of-Planned-Behavior-informed model provided a better fit. In the model, social cognitions fully mediated the relationships from the activity facet and industriousness to intentions for and engagement in physical activity, such that the relationships were primarily maintained by positive affective evaluations, positive expected outcomes, and confidence in overcoming barriers related to physical activity engagement. The resultant model – termed the *Disposition-Belief-Motivation model*– is proposed as a useful framework for organizing and integrating personality trait facets and social cognitions from various theoretical perspectives to investigate the expression of health-related behaviors, such as physical activity. Moreover, the results are discussed in terms of extending the application of the Disposition-Belief-Motivation model to longitudinal and intervention designs for physical activity engagement.

## Introduction

Accumulated research findings demonstrate the importance of social cognitions – beliefs, attitudes, and values anchored in the context of exercise – as correlates and predictors of physical activity intentions and behavior (e.g., Hausenblas et al., 1997; Courneya et al., 2006; Scholz et al., 2009; Rhodes et al., 2010; Lee, 2011; Poomsrikaew et al., 2012; White et al., 2012). Increasingly, research also has incorporated personality traits, especially from the Big Five domains of extraversion and conscientiousness, into social cognition models to account for the effects of these relatively stable *trans*-situational dispositional tendencies on physical activity intentions and physical activity behavior (e.g., Rhodes et al., 2002, 2004b; Rhodes and Courneya, 2003; Bogg, 2008; Hoyt et al., 2009). Findings from this research have shown social cognition variables from disparate theoretical traditions – including the Theory of Planned Behavior (Ajzen, 1991), Social Cognitive Theory (Bandura, 1977, 1986), and the Transtheoretical Model of Change (Prochaska and DiClemente, 1983) – serve to maintain (i.e., mediate), in part, relations between traits and physical activity behavior.

Importantly, none of this work has bridged across these three major social cognition frameworks to investigate the structure of the multifarious components of these models, let alone incorporate key physical activity-related personality trait facets into such an integration, such as trait activity (a facet of extraversion) and industriousness (a facet of conscientiousness). Prior research suggests there may be an underlying structure linking these trait facets, exercise social cognitions, physical activity intentions, and physical activity behavior. However, currently, there is no comprehensive framework that attempts to organize trait and social cognition influences on physical activity intentions and engagement. Using two distinct theoretical perspectives – the Theory of Planned Behavior and Neo-Socioanalytic Theory – the primary aim of the present work is to test two structural models of constructs from these varied levels of analysis and conceptual origins using a representative U.S. sample.

### The Scope of Theory of Planned Behavior, Social Cognitive Theory, and Transtheoretical Model Variables’ Associations with Physical Activity

Perceived behavioral control, attitudes, subjective norms, and behavioral intentions are the primary constructs from the Theory of Planned Behavior (Ajzen, 1991). Perceived behavioral control is the extent to which an individual believes she/he can control a behavior at will. Attitudes are the affective (emotion-laden) and instrumental (relating to benefits and costs) evaluations associated with the performance and consequences of the behavior. Subjective norms are the perceptions of influence an individual feels from the social environment (typically, close others) to either refrain from a behavior or engage in a behavior. Greater perceived behavioral control, endorsement of positive attitudes, and greater perceptions of social endorsement influence the formulation of intentions, which are general goals related to an individual’s planned level of engagement in a behavior.

Meta-analytic and primary research findings have shown that attitudes directly predict physical activity intentions and perceived behavioral control predicts both intentions and actual physical activity behavior (Hausenblas et al., 1997; Rhodes et al., 2010; Lee, 2011). When attitudes were separated into affective and instrumental components, research showed instrumental attitudes did not significantly predict physical activity intentions or behavior, while affective attitudes showed strong, positive relations to intentions and behavior (Lowe et al., 2002; Rhodes et al., 2005). In the present study, we exclude instrumental attitudes from consideration due to their lower predictive validity for physical activity intentions and behavior in past research, as well as to avoid redundancy with the outcome expectancies construct from Social Cognitive Theory (Bandura, 2004). Although several studies have shown that subjective norms predict physical activity intentions (e.g., Rhodes et al., 2004b; Latimer and Ginis, 2005; Lee, 2011), few have shown prediction of physical activity behavior by subjective norms. More specifically, Okun et al. (2002) showed that only friend (as opposed to family) descriptive norms (one of three types of subjective norms examined in the study) were predictive of physical activity behavior. Subjective norms are included in the present study in order to assess all Theory of Planned Behavior variables and to clarify the relationship between norms and physical activity in a large, representative sample.

Two constructs from Social Cognitive Theory (Bandura, 1977, 1986), outcome expectancies and self-efficacy, also have been examined in conjunction with physical activity behavior. Outcome expectancies are beliefs about the likelihood of positive and negative consequences (as opposed to positive or negative evaluations, i.e., attitudes) of a behavior (Bandura, 1986; Wójcicki et al., 2009). Self-efficacy is an individual’s level of confidence in being able to perform a behavior in the face of challenges and/or obstacles (Bandura, 1977). Aside from its integration with tests of the Transtheoretical Model (e.g., Bogg, 2008), meta-analytic findings showing links between self-efficacy and physical activity behavior and Theory of Planned Behavior variables have spurred calls for self-efficacy to be more systematically integrated into the Theory of Planned Behavior (Hagger et al., 2002). In several other studies, self-efficacy and outcome expectancies have shown significant direct and indirect effects on physical activity intentions and behavior across a variety of samples and nations (Brassington et al., 2002; Scholz et al., 2009; Poomsrikaew et al., 2012; White et al., 2012). These findings highlight the contributions of self-efficacy and outcome expectancies to physical activity intentions and behavior and provide evidence to support their inclusion in a structural integration of trait and social cognition influences on physical activity.

In the Transtheoretical Model, there are five behavioral processes of change that are postulated as means of enacting intentions, including counter-conditioning, helping relationships, reinforcement management, self-liberation, and stimulus control (Prochaska and DiClemente, 1983). The Transtheoretical Model also includes five cognitive processes of change and decisional balance, but these constructs are excluded from the present study due to their redundancy with attitudes, outcome expectancies, and/or norms.

### The Role of Personality Traits in Physical Activity Behavior and Physical-Activity-Related Social Cognitions

Two personality trait facets – the conscientiousness-related facet of industriousness and the extraversion-related facet of activity – have shown the most consistent and strongest associations with physical activity behavior (e.g., Conner and Abraham, 2001; Rhodes and Courneya, 2003; Rhodes et al., 2005; Bogg, 2008; Rodgers et al., 2008; Hoyt et al., 2009). Individuals scoring high on the activity facet are characterized as being busy, on the go, and occupied (Costa and McCrae, 2008). In several examinations in conjunction with the Theory of Planned Behavior, the activity facet was found to have direct effects on physical activity attitudes and perceived behavioral control (Rhodes and Courneya, 2003; Hoyt et al., 2009), exhibit direct and indirect effects on intentions through perceived behavioral control and attitudes (Hoyt et al., 2009), and moderate the relationship between intention and of Theory of Planned Behavior variables (Rhodes et al., 2005). These studies used both healthy student samples and samples of breast, prostate, colon, and lung cancer survivors.

In meta-analytic work, industriousness was found to be one of the strongest conscientiousness-related predictors of physical activity behavior (Bogg and Roberts, 2004). Individuals who score high on the industriousness facet are characterized as being hard-working, tenacious, resourceful, ambitious, and confident (Roberts et al., 2004, 2005). Industrious individuals report greater perceived behavioral control, endorse subjective physical activity norms and attitudes, exhibit more consistent physical activity intention-behavior relations (Rhodes et al., 2005), show greater self-efficacy in overcoming barriers, and report more frequent use of the behavioral processes of change (Bogg, 2008).

Taken together, the above findings provide evidence for the importance of trait activity and industriousness as physical activity-relevant individual difference factors that predict physical activity-related social cognitions, as well as physical activity behavior in various populations.

### Testing Theory of Planned Behavior and Neo-Socioanalytic Theory Structural Models of Trait and Social Cognition Influences on Physical Activity

Despite the reported relations in the extant literature, integrations of traits, social cognitions, and physical activity behavior across models are still scarce, even though the relationships from these theoretical perspectives appear to be complementary and suggestive of a larger set of psychological influences on physical activity. Using a representative sample of adults, the present study sought to expand this body of research by testing integrated models of the relationships among trait facets of activity and industriousness; social cognitions that include perceived behavioral control, norms, affective attitudes, outcome expectancies, self-efficacy, behavioral processes of change, and intentions; and the discrete behavior of physical activity.

We investigated these relationships using an alternative-models comparison of a Theory-of-Planned-Behavior-informed arrangement and a Neo-Socioanalytic-Theory-informed arrange-ment of the constructs. The constructs were selected for inclusion based upon the following criteria: (1) The construct represented a core component of the Theory of Planned Behavior, Social Cognitive Theory, or Transtheoretical Model frameworks; (2) Prior work demonstrated a relationship between the construct and other exercise social cognitions, physical activity intentions, physical activity behavior, or trait activity or industriousness; and (3) The construct showed conceptual independence (i.e., non-overlap in construct content) from other core components of the Theory of Planned Behavior, Social Cognitive Theory, and/or Transtheoretical Model frameworks. Across both models, it was expected that traits would be the most distal influences on physical activity behavior, as it is generally assumed that traits are among the background factors that influence social cognitions and subsequent behaviors (Ajzen, 2011). However, the variation across the two models lies in the arrangement of the varying levels of abstraction and the direct and indirect paths among the seven social cognition constructs mentioned above.

### A Theory-of-Planned-Behavior-Informed Structure of Trait and Social Cognition Influences on Physical Activity

**FIGURE 1:**
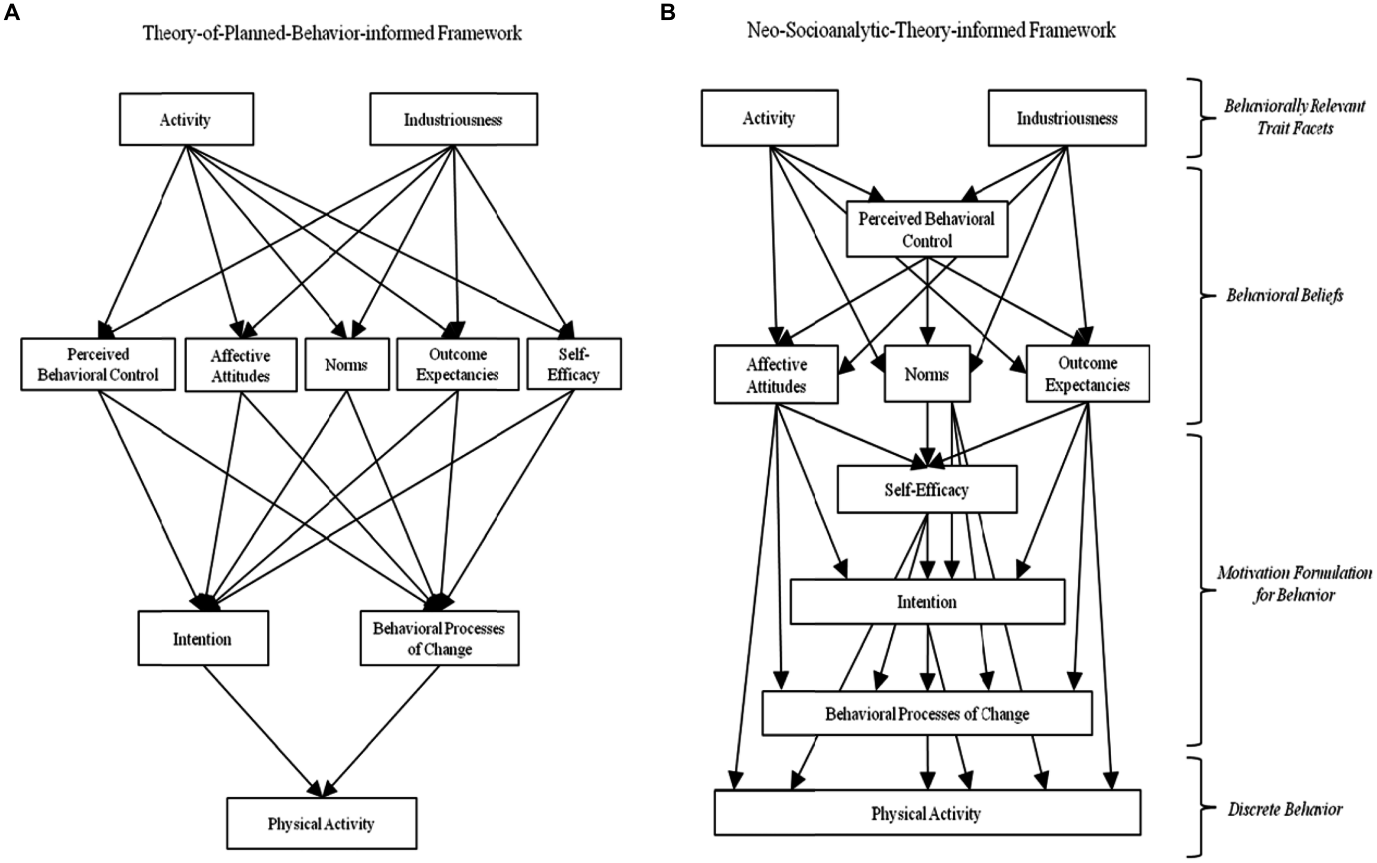
**(A)** Depicts the hypothesized theoretical arrangement of the Theory-of-Planned-Behavior-informed framework. **(B)** Depicts the hypothesized theoretical arrangement of the Neo-Socioanalytic-Theory-informed framework.

The Theory of Planned Behavior is one of the most widely used psychological models for explaining various health-related behaviors and, as noted above, some researchers have integrated Theory of Planned Behavior constructs with trait activity and industriousness to further elaborate psychological influences on physical activity behavior. As such, and given its inclusion of three disparate social cognition constructs, the Theory of Planned Behavior represents a candidate conceptual substrate upon which other social cognition constructs could be added. A core postulate of the Theory of Planned Behavior is that perceived behavioral control, norms, and attitudes all reside at the same level of abstraction (i.e., none of these three constructs are considered to be antecedent to any of the others) in their predicted effects on intention (which, in turn, predicts behavior; Ajzen, 1991). In line with the Theory of Planned Behavior assumption of conceptual equivalence, the Social Cognitive Theory constructs of self-efficacy and outcome expectancies are expected to reside at the same level as perceived behavioral control, norms, and affective attitudes (see **Figure 1A**).

Bandura’s (2004) assertion that goals are composed of (A) the motivation (i.e., intention) to enact the goal *and* (B) the actual strategies used to engage in the behavior suggests behavioral processes of change reside at the same level of abstraction as intentions; the assumption being that intentions and behavioral processes of change are both markers of the larger goal construct. Given the arrangement of this Theory-of-Planned-Behavior-informed structural model, it was expected that trait activity and industriousness would predict perceived behavioral control, norms, affective attitudes, self-efficacy, and outcome expectancies. In turn, perceived behavioral control, affective attitudes, norms, outcome expectancies, and self-efficacy would predict intentions and the behavioral processes of change, which would predict physical activity behavior (see **Figure 1A**).

Although the original Theory of Planned Behavior model did not explicate indirect effects among social cognition constructs and actual behavior, it was expected that there would be mediation effects where there are intervening levels of abstraction, such that perceived behavioral control, affective attitudes, norms, outcome expectancies, and self-efficacy would mediate the relationships between trait facets and physical activity intentions and behavioral processes of change. In turn, the relationships between social cognitions and physical activity behavior were expected to be mediated by physical activity intentions and behavioral processes of change. These expectations are consistent with previous research that showed significant indirect effects among these constructs (e.g., Hoyt et al., 2009; Poomsrikaew et al., 2012), as well as the Theory of Planned Behavior postulate that personality permeates and guides thoughts and actions (Ajzen, 2011).

### A Neo-Socioanalytic-Theory-Informed Structure of Trait and Social Cognition Influences on Physical Activity

An alternative model for the integration of trait and social cognition influences on physical activity is Neo-Socioanalytic Theory (Roberts and Wood, 2006), which has been utilized in previous integrations of traits and social cognitions with health-related behaviors, including physical activity (Bogg, 2008; Bogg et al., 2008). Neo-Socioanalytic Theory posits several levels of contextual abstraction within four domains of individual differences (traits, motives/values, abilities, and narratives) that can be organized hierarchically according to their psychological proximity to one another, where psychological proximity is the theoretically anticipated strength of relations among constructs. Among a given set of constructs, this means psychological proximity is informed by the presence of conceptually meaningful shared features (especially the content and context of the constructs; Bogg, 2008). Accordingly, a Neo-Socioanalytic Theory perspective would locate trait activity and industriousness at the most decontextualized (i.e., *trans*-situational) level of abstraction and thus, most distal from the discrete level of physical activity behavior (see **Figure 1B**).

In contrast to the Theory-of-Planned-Behavior-informed model, perceived behavioral control resides alone below the trait level, due to it being the broadest form of beliefs about one’s ability to engage in physical activity. That is, perceived behavioral control is entirely devoid of evaluative weighting, explicit outcome forecasting, and/or considerations of internal and/or external forms of support or hindrance for physical activity (cf. Trafimow et al., 2002). In the Neo-Socioanalytic-Theory-informed model, affective attitudes, norms, and outcome expectancies are located below perceived behavioral control as these constructs embed beliefs tied to explicit outcome forecasting, evaluative weighting, and considerations of internal and/or external forms of support or hindrance related to physical activity behavior. Below affective attitudes, norms, and outcome expectancies are motivational formulations about the behavior, which include self-efficacy and further down, intentions, to engage in physical activity. Self-efficacy was expected to be more distal from discrete behavior than intentions because it pertains to individuals’ confidence in engaging in the behavior despite specific barriers, which would require the recollection of past experiences of physical activity in specific situations and/or imagining the effects of circumstances that have not yet been experienced. In contrast, intentions signify a specific level of planned commitment to actual engagement in physical activity behavior. Behavioral processes of change (i.e., behavioral enaction techniques and strategies) were expected to reside below the level of intentions owing to their conceptualization as means by which individuals translate intentions into behavioral engagement. Finally, below the behavioral processes of change would be the level of behavioral enaction, i.e., physical activity behavior.

Based on psychological proximity and past research, it was expected that activity and industriousness would be directly and indirectly related to physical activity behavior via intervening social cognition constructs (e.g., Rhodes et al., 2003; Hoyt et al., 2009). Individuals who scored high in activity were expected to hold positive physical activity attitudes and outcome expectancies, as well as report support from their environment (i.e., norms) to engage in physical activity. Moreover, because individuals high on activity are typically active and on the move, they were expected to feel more self-efficacious about physical activity, endorse greater use of the behavioral processes of change, and engage in more physical activity due to their natural propensity to be active. Individuals high in industriousness were expected to hold more positive physical activity outcome expectancies, attitudes, and norms due to their general tendencies to be hard-working and achievement-oriented. Individuals high in industriousness also were expected to have greater physical activity self-efficacy, stronger intentions, and endorse more frequent use of the behavioral processes of change due to their generally greater levels of tenacity. Neo-Socioanalytic Theory suggests constructs at each level of abstraction would be directly and indirectly related to variables at lower levels of abstraction. Therefore, we expected a cascade of interrelated influences among perceived behavioral control, norms, affective attitudes, outcome expectancies, self-efficacy, intentions, behavioral processes of change, and physical activity behavior (see **Figure 1B**), such that as the constructs shift from the most decontextualized level of traits to the discrete level of behavior, each domain becomes more deeply embedded in the behavioral context and stronger direct and indirect associations would be expected.

Provided that both models show adequate internal fit with the data, a test of these competing models would help identify the underlying structure of the relations among traits and social cognition influences on physical activity intentions and behavior. Additionally, the use of a large, nationally representative sample bolsters confidence that the final identified model could be useful in organizing the disparate relations found in previous research and might inform future descriptive and intervention research in physical activity behavior.

## Materials and Methods

### Participants

The survey was conducted using the Web-enabled KnowledgePanel^®^, a probability-based national panel designed to be representative of the U.S. population. Initially, participants were chosen scientifically by a random selection of telephone numbers and residential addresses. Persons in selected households were then invited by telephone or by mail to participate. For those who agreed to participate, but did not already have Internet access, GfK provided a laptop and ISP connection at no cost. People who already had computers and Internet service were permitted to participate using their own equipment. Panelists then received unique log-in information for accessing surveys online (completion rate: 63.4 %). For a more thorough explanation and justification for the representativeness of the KnowledgePanel^®^sample, interested readers can follow this link: *http://www.knowledgenetworks.com/ganp/docs/KnowledgePanelR-Statistical-Methods-Note.pdf.* The study was approved by the Wayne State University’s Institutional Review Board with exempt status. Participants were consented by GfK as members of the KnowledgePanel^®^. In the present study, participants (*N* = 957) ranged from 18 to 88 years of age, with a mean age of 49.61 years (SD = 16.97 years). The sample was sex-balanced (50.3% females and 49.7% males) and the majority of the participants were White, Non-Hispanic (75.1%).

Sampling weights were used in all analyses to modify the sample characteristics to be representative of the U.S. population. The weighting procedure adjusted for survey non-response, as well as non-coverage or under- or over-sampling or participant demographic factors. Specifically, the weighting procedure adjusted for the demographic factors of sex, age, race/Hispanic ethnicity, education, census region, household income, residence in a metropolitan area, and Internet access.

### Trait, Social Cognition, and Physical Activity Assessment Materials

#### Activity Facet

The extraversion-related facet of activity was assessed using an International Personality Item Pool analog scale of the NEO-Personality Inventory-Revised activity scale (Goldberg et al., 2006). Participants rated 10 items using a five-point Likert scale. (e.g., “is always on the go, is always busy, [or] does a lot in my spare time.”; 1 = Disagree strongly, 5 = Agree strongly; α = 0.69).

#### Industriousness Facet

Five adjectives [e.g., industrious, thorough, tenacious, thrifty, lazy (reverse-scored)] were used to assess the conscientiousness-related facet of industriousness (Roberts et al., 2004). Participants rated the items using a five-point Likert scale (1 = Very uncharacteristic, 5 = Very characteristic; α = 0.67).

#### Affective Attitudes

Four items were used to measure affective physical activity attitudes using bipolar semantic differential adjectives on a seven-point scale (Courneya and Bobick, 2000). The items were enjoyable–unenjoyable, boring–interesting, pleasant–unpleasant, stressful–relaxing (α = 0.91).

#### Subjective Norms

Three items were used to measure participants’ perceptions about the beliefs of close associates regarding regular exercise behavior on a seven-point Likert scale (1 = Strongly disagree, 7 = Strongly agree; α = 0.91; Courneya et al., 1999). The items are: People who are important to me: (1) think I should participate in regular exercise; (2) encourage me to participate in regular exercise; and (3) support me in participating in regular exercise.

#### Perceived Behavioral Control

Perception of control in physical activity engagement was assessed using three items (Ajzen and Madden, 1986; Courneya and Bobick, 2000; α = 0.85). The first item asked participants: “How much control do you have over participating in regular exercise?” and was rated on a five-point Likert scale (1 = Very little control, 5 = Complete control. The other two items were: “If I wanted to, I could easily participate in regular exercise” and “How much I participate in regular physical exercise is completely up to me.” These items were also rated on a five-point Likert scale (1 = Strongly disagree, 5 = Strongly agree).

#### Self-Efficacy

Confidence regarding abilities to overcome obstacles or challenges related to performing physical activity was assessed using an 18-item exercise self-efficacy scale (Benosovich et al., 1998). Each item assessed confidence in being able to “participate in regular exercise.” Example items include, “I have to exercise alone,” “I am busy,” “I am spending time with friends or family who do not exercise,” “I am traveling,” “I am anxious,” and “It’s cold outside.” The items were rated on a five-point Likert scale (1 = Not at all confident, 5 = Extremely confident; α = 0.96).

#### Outcome Expectancies

Eleven items from the multidimensional outcome expectations for exercise scale (Wójcicki et al., 2009), as well as three items from the outcomes expectancies questionnaire (Waters et al., 2012), were used to assess positive and negative expectations related to exercise. Example items include: “Exercise will improve my ability to perform daily activities” and “I can hurt myself if I exercise regularly (reverse-scored).” Items were rated on a five-point Likert scale (1 = Strongly disagree, 5 = Strongly agree; α = 0.82).

#### Intention

Intention to engage in physical activity was measured with a single open-ended item (Courneya, 1994). The item read: “I plan to participate in physical exercise at least _____ times per week every week,” and participants reported how many times they planned to exercise within the specified time.

#### Behavioral Processes of Change

Fifteen items (three for each of the five behavioral processes of change) assessed use of behavioral processes of change (i.e., counter-conditioning, helping relationships, reinforcement management, self-liberation, and stimulus control) to engage in physical activity (Nigg et al., 1998). Counter-conditioning is the replacement other less active behaviors, such as sleeping or watching television, with physical activity. Helping relationships refers to the recruitment and utilization of support from close others to engage in physical activity. Reinforcement management is the use of reminders related to the reward of engaging in physical activity. Self-liberation describes efforts at committing to the belief that one can engage in physical activity. Stimulus control is the (re-)organization of situations and cues that foster engagement in physical activity. Nigg et al. (1998) validated these processes of change in the context of physical activity. Validity of the processes of change scales for physical activity has also been tested in several different populations, such as adolescents (Rhodes et al., 2004a), college students (Dishman et al., 2010), and older adults (Gorely and Gordon, 1995). Items were rated on a five-point Likert scale (1 = Never, 5 = Repeatedly; α = 0.85). The instructions stated: “The following experiences can affect the exercise habits of some people. Think of similar experiences you may be currently having or have had during the past month. Then rate how frequently the event occurs by circling the appropriate number.” Example items are: “I make sure I always have a clean set of exercise clothes, I have someone who encourages me to exercise, [and] I make commitments to exercise.”

#### Physical Activity

The Godin Leisure-Time Exercise Questionnaire (Godin and Shephard, 1985) was used to assess the frequency of engagement in strenuous, moderate, and mild physical activity on a weekly basis. The strenuous exercise item assessed the number of times participants engaged in more than 20 min of exercise that causes the heart to beat rapidly (e.g., running, football, vigorous swimming) during a typical 7-days period. The moderate exercise item assessed the number of times participants engaged in more than 20 min of exercise that requires effort but is not exhausting (e.g., fast walking, volleyball, social dancing) during a typical 7-days period. The mild exercise item was excluded from analyses because recommendations from the U.S. Department of Health and Human Services and the American College of Sports Medicine emphasize the health-related benefits of moderate and vigorous physical activity (U.S. Department of Health and Human Services [U.S. HHS], 2008; Garber et al., 2011). The number of times participants reported engaging in vigorous and moderate forms of physical activity were summed to create a composite score of physical activity.

### Analytic Approach

Path models were constructed according to the hypothesized arrangement (**Figure 1**) of the variables according to each theoretical framework and analyzed (via Amos v.22) using a variance-covariance matrix to incorporate sampling weights. Bootstrapping procedures (*k* = 5,000) were used to test for indirect effects, as indicated by 95% confidence intervals around the estimates of indirect effects that did not include zero (MacKinnon et al., 2007; Cheong and MacKinnon, 2012; Hancock and Liu, 2012). Internal model fit was assessed through an examination of standardized path weights that were statistically significant (*p* < 0.05), the root mean square error of approximation (RMSEA), and the comparative fit index (CFI). RMSEA measures the closeness of fit of a model in relation to its degrees of freedom and values that are close to zero indicate good fit (Browne and Cudeck, 1993). If RMSEA is less than or equal to 0.05, this indicates adequate fit. Greater CFI scores (ranging from 0 to 1) indicate better fit. A model with a CFI of 0.90 or greater (meaning that at least 90% of the covariation in the data is reproduced by the model) indicates adequate fit (Bentler, 1990).

Provided that both models showed good internal fit to the data, they were then compared using the Bayesian information criterion (BIC) and the Akaike information criterion (AIC) to determine which of the two models showed better comparative fit. The BIC and AIC both help identify which model reproduces the observed variances and covariances using the fewest parameters (i.e., with greater parsimony). The BIC is interpreted as an odds ratio, whereby a lower BIC value indicates better comparative fit (Raftery, 1995). Specifically, given two models with a difference of 10 points, this indicates that the odds are approximately 150:1 that the model with the lower BIC value provides a better comparative fit than the model with the higher BIC value (Raftery, 1995). Although not interpreted as odds, lower AIC values also indicate better comparative fit (Akaike, 1987). The calculation of BIC incorporates a weighting that results in a comparatively strong penalty for greater model complexity, whereas the calculation of the AIC does not overweight for model complexity.

## Results

### Descriptive Statistics and Correlations

**Table 1** shows the descriptive statistics and correlations for the study variables. The only non-significant correlation found was that between the activity facet and subjective norms. All other correlations were significant at *p* < 0.01.

**Table 1:** Descriptive statistics and correlations among study variables.

	1	2	3	4	5	6	7	8	9	10
(1) Activity facet	–									
(2) Industriousness facet	0.34	–								
(3) Affective attitudes	0.22	0.20	–							
(4) Norms	0.04_ns_	0.12	0.30	–						
(5) Perceived behavioral control	0.12	0.29	0.36	0.33	–					
(6) Barriers self-efficacy	0.17	0.24	0.45	0.28	0.30	–				
(7) Outcome expectancies	0.11	0.28	0.51	0.43	0.45	0.40	–			
(8) Intention	0.11	0.19	0.48	0.29	0.33	0.45	0.40	–		
(9) Behavioral processes of change	0.17	0.24	0.59	0.46	0.38	0.56	0.59	0.61	–	
(10) Physical activity (moderate/strenuous)	0.13	0.11	0.37	0.22	0.23	0.33	0.29	0.64	0.50	–

Mean	3.08	3.82	3.72	3.54	4.21	2.77	3.6	2.91	2.66	2.9
SD	0.54	0.64	1.04	1.03	0.92	0.99	0.57	2.11	0.85	3.16

*_ns_, non-significant. All other correlations are significant at *p* < 0.01.*

### Model Comparisons

**FIGURE 2:**
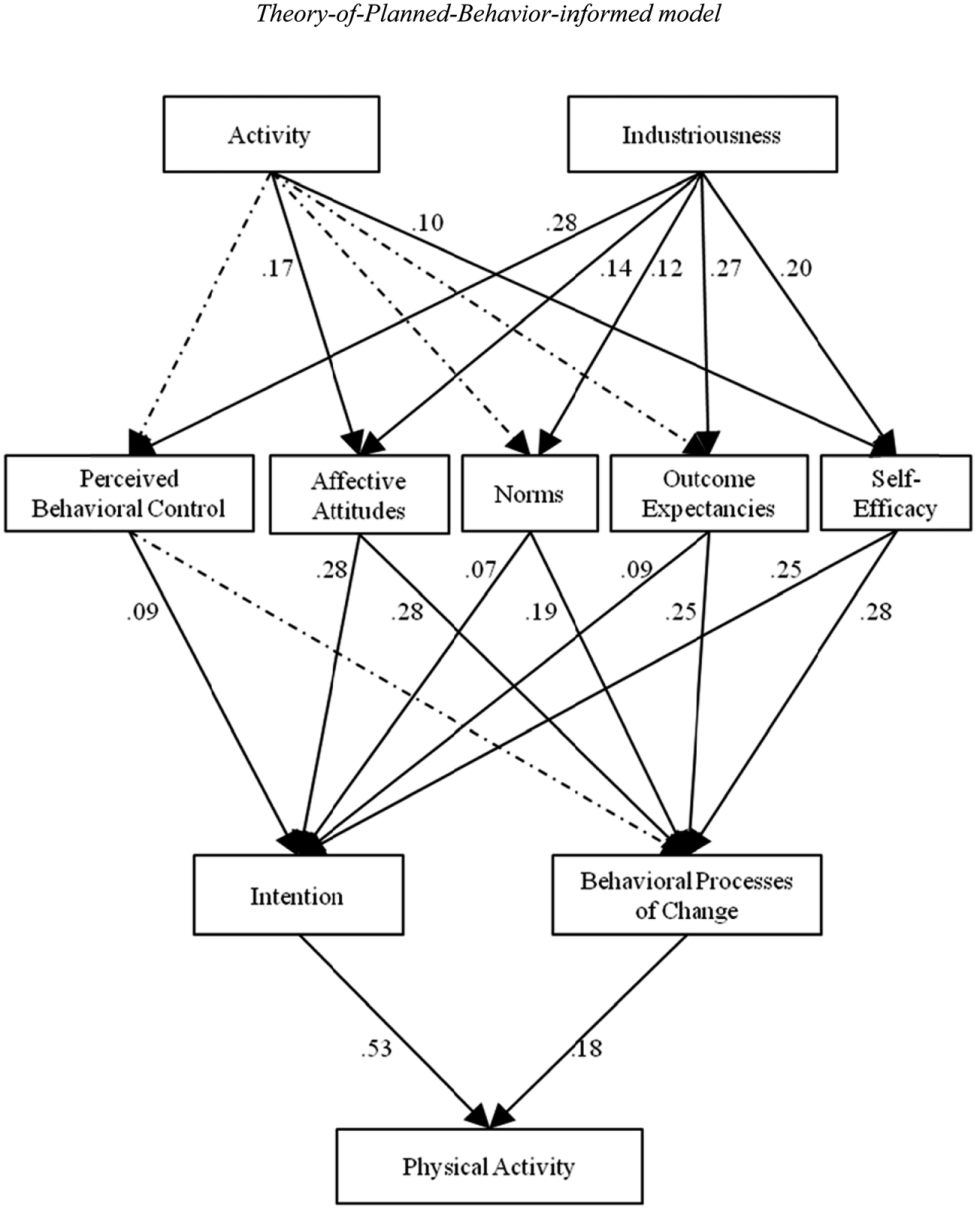
**Direct standardized path weights of the Theory-of-Planned-Behavior-informed model.** Dotted lines represent non-significant paths, for which the path weights were omitted. All other lines and path weights shown are statistically significant at *p* < 0.01. Correlated error terms were also omitted for clarity of presentation, and they are as follows: activity ↔ industriousness = 0.12; perceived behavioral control ↔ affective attitudes = 0.29; affective attitudes ↔ norms = 0.30; norms ↔ outcome expectancies = 0.23; outcome expectancies ↔ self-efficacy = 0.19; perceived behavioral control ↔ norms = 0.28; affective attitudes ↔ outcome expectancies = 0.27; norms ↔ self-efficacy = 0.26; perceived behavioral control ↔ outcome expectancies = 0.19; affective attitudes ↔ self-efficacy = 0.40; perceived behavioral control ↔ self-efficacy = 0.21; and intention ↔ behavioral POCs = 0.35.

**FIGURE 3:**
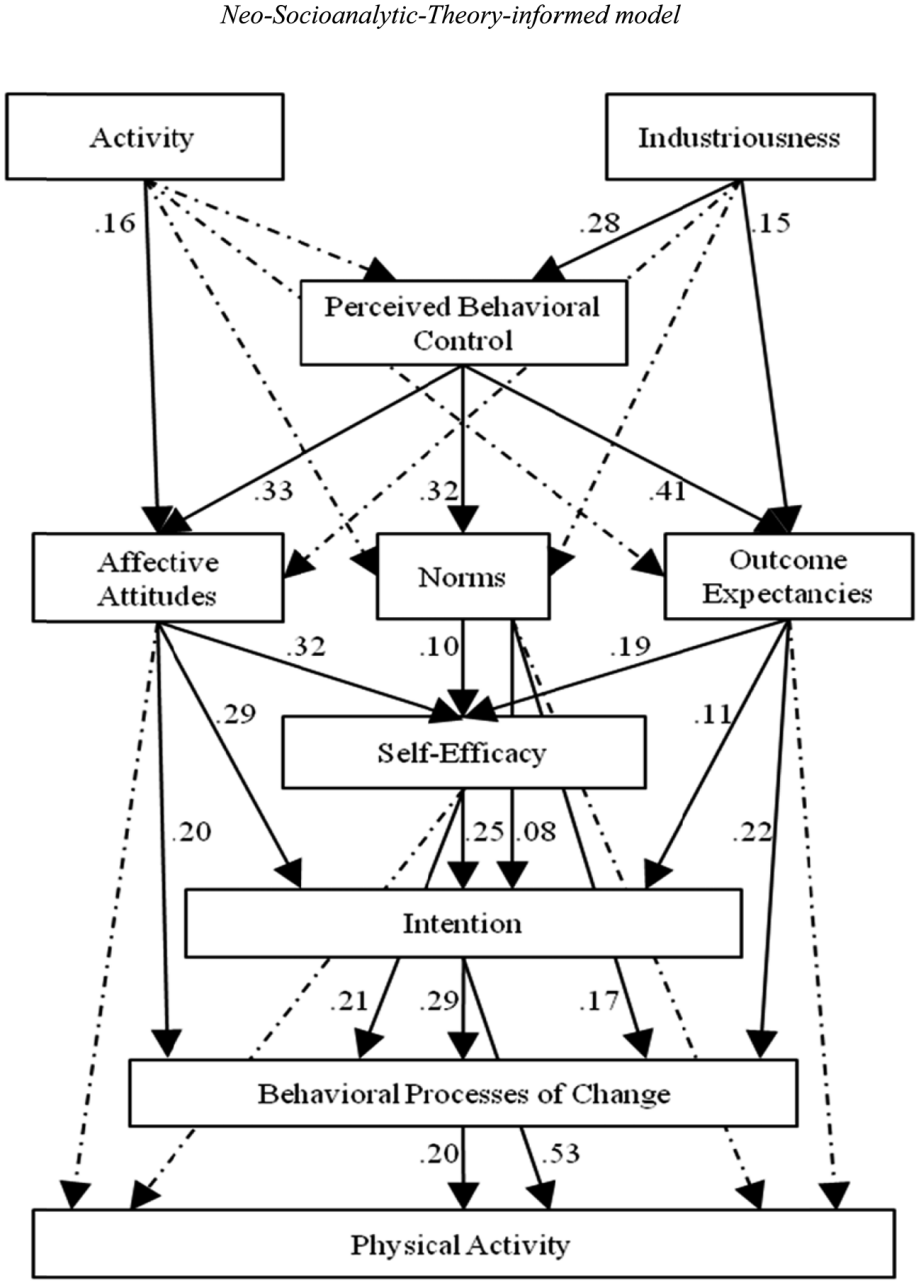
**Direct standardized path weights of the Neo-Socioanalytic-Theory-informed model.** Dotted lines represent non-significant paths, for which the path weights were omitted. All other lines and path weights shown are statistically significant at *p* < 0.01. Correlated error terms were also omitted for clarity of presentation, and they are as follows: activity ↔ industriousness = 0.34; affective attitudes ↔ norms = 0.21; norms ↔ outcome expectancies = 0.33; affective attitudes ↔ outcome expectancies = 0.41.

**Table 2** shows the fit statistics and indices for the Theory-of-Planned-Behavior-informed model and the Neo-Socioanalytic-Theory-informed model. As can be seen, both models had a CFI score of at least 0.99, indicating that at least 99% of the covariation in the data was reproduced by each model. The models also showed acceptable errors of approximation, with RMSEA at 0.016 for the Theory-of-Planned-Behavior-informed model and at 0.051 for the Neo-Socioanalytic-Theory-informed model. As indicated by the lower BIC and lower AIC values, the Theory-of-Planned-Behavior-informed model provided a better comparative fit than the Neo-Socioanalytic-Theory-informed model. Subsequent interpretation of the data references the Theory-of-Planned-Behavior-informed model. **Figure 2** shows the standardized path weights for the direct effects in this model. For comparison purposes, **Figure 3** shows the standardized path weights for the direct effects in the Neo-Socioanalytic-Theory-informed model.

**Table 2:** Internal and comparative fit statistics for Theory-of-Planned-Behavior-informed and Neo-Socioanalytic-Theory-informed models.

	χ^2^	*df*	*p*	*r*^2^	RMSEA	CFI	BIC	AIC
Theory-of-Planned-Behavior-informed Framework	13.573	11	0.258	0.43	0.016	0.999	315.118	101.573
Neo-Socioanalytic-Theory-informed Framework	41.574	12	0.000	0.43	0.051	0.990	336.266	127.574

*RMSEA, root mean square error of approximation; CFI, comparative fit index; BIC, Bayesian information criterion; AIC, Akaike information criterion.*

### Theory-of-Planned-Behavior-Informed Model of Direct and Indirect Effects

As shown in **Figure 2**, path analyses indicated that individuals who scored higher on industriousness were more likely to report greater behavioral control, endorse positive affective attitudes, norms, and outcome expectancies, and feel more confident in overcoming barriers to exercise. Individuals who scored higher on the activity facet endorsed more positive affective attitudes and reported greater self-efficacy, but did endorse greater perceived behavioral control, norms, and outcome expectancies. Predictions from the original Theory of Planned Behavior model held in the expanded model, such that perceived behavioral control, affective attitudes, and norms all significantly predicted intentions. Outcome expectancies and self-efficacy also showed significant relations with intentions. Affective attitudes, norms, outcome expectancies, and self-efficacy significantly predicted behavioral processes of change. Finally, both intentions and behavioral processes of change showed significant relations to physical activity behavior.

Additionally, constructs in the model showed indirect effects (at *p* < 0.01) to other constructs where there were intervening levels of abstraction. Both trait facets showed indirect effects to intention (activity facet β = 0.08; industriousness facet β = 0.15), behavioral processes of change (activity facet β = 0.08; industriousness facet β = 0.19), and physical activity (activity facet β = 0.05; industriousness facet β = 0.11). Affective attitudes (β = 0.20), norms (β = 0.07), outcome expectancies (β = 0.09), and self-efficacy (β = 0.18) showed indirect effects to physical activity through both intention and behavioral processes of changes, while perceived behavioral control (β = 0.05) showed indirect effects to physical activity through intention alone. **Table 3** displays the SE and lower and upper bounds of the 95% confidence intervals for these effects.

**Table 3:** Standardized indirect effects of Theory-of-Planned-Behavior-informed path model.

	Activity facet	Industriousness facet	Perceived behavioral control	Affective attitudes	Norms	Outcome expectancies	Self-efficacy
	β (SE)	β (SE)	β (SE)	β (SE)	β (SE)	β (SE)	β (SE)
	(95% CI)	(95% CI)	(95% CI)	(95% CI)	(95% CI)	(95% CI)	(95% CI)
Behavioral processes of change	0.08 (0.03)	0.19 (0.03)	–	–	–	–	–
	(0.03, 0.13)	(0.14, 0.24)					
Intention	0.08 (0.02)	0.15 (0.02)	–	–	–	–	–
	(0.04, 0.11)	(0.11, 0.19)					
Physical activity (moderate/strenuous)	0.05 (0.02)	0.11 (0.02)	0.05 (0.02)	0.20 (0.02)	0.07 (0.02)	0.09 (0.02)	0.18 (0.02)
	(0.03, 0.08)	(0.08, 0.14)	(0.01, 0.09)	(0.16, 0.24)	(0.04, 0.11)	(0.05, 0.13)	(0.14, 0.22)

*All bootstrapped 95% confidence intervals exclude zero (0) and all indirect effects are statistically significant at *p* < 0.01. Dashes (–) indicate the absence of an intervening variable through which an indirect effect could be computed.*

## Discussion

Using a representative U.S. sample, the primary aim of the present study was to test alternative theoretical organizations of the multifarious personality trait and social cognition influences on physical activity. Guided by prior research and insights from the Theory of Planned Behavior and Neo-Socioanalytic Theory, the extraversion-related facet of activity, the conscientiousness-related facet of industriousness, constructs from the Theory of Planned Behavior (affective attitudes, subjective norms, perceived behavioral control, intentions), Social Cognitive Theory (self-efficacy, instrumental outcome expectancies), the Transtheoretical Model (behavioral processes of change), and physical activity were modeled using two different approaches. Both the Theory-of-Planned-Behavior-informed model and the Neo-Socioanalytic-Theory-informed model showed that the integration of these influences in a comprehensive framework is fruitful (as both models showed good internal fit to the data) and can provide valuable insights into additional relations of interest among these constructs. Moreover, the comparative model fit indices favored the Theory-of-Planned-Behavior-informed model over the Neo-Socioanalytic-Theory-informed model, providing support for a less stratified structure – a *Disposition-Belief-Motivation* model of these influences on physical activity behavior.

### Evidence for an Integrative Disposition-Belief-Motivation Model of Physical Activity

The pathways identified in the Disposition-Belief-Motivation model are mostly consistent with prior research that integrated traits into social cognition models, but also elucidated new relationships that have not been previously explored. Specifically, individuals who reported more positive affective attitudes, norms, and outcome expectancies toward physical activity behavior and greater confidence in overcoming barriers to physical activity behavior reported more use of the behavioral processes of change – relationships that have not been tested in previous research. Contrary to past research findings (e.g., Rhodes and Courneya, 2003; Rhodes et al., 2004b), significant paths from the activity facet to perceived behavioral control and norms were not found. This discrepancy may be due to sampling differences. Previous studies often relied upon female-skewed samples and/or samples characterized by a specific form of morbidity. The present study provides novel findings related to facet-specific pathways to engagement in physical activity, and confidence in these findings is bolstered by the use of a large sample that is representative of not only sex, but also age, ethnicity, income, education, type and region of residence, and Internet access.

Interestingly, a path from perceived behavioral control to behavioral processes of change was not found. It is possible that individuals who perceive themselves as having greater perceived control over physical activity behavior do not feel the need to utilize behavioral strategies due to a greater belief in the ability to directly execute physical activity actions. However, as suggested by the small indirect effect of perceived behavioral control on physical activity behavior, perceived control does not signify actual control over behavior. Future research should explore the extent to which perceived control over physical activity behavior – in the absence of plans and strategies – would lead to actual physical activity behavior. The evidence from the present work suggests this direct pathway is unlikely.

Although the definitions of perceived behavioral control and self-efficacy overlap to some degree, it has been argued that these constructs can be reliably distinguished from each other in predictions of intentions and behaviors (e.g., Trafimow et al., 2002). In the present work, the self-efficacy measure assessed confidence in overcoming barriers to physical activity behavior, whereas the perceived behavioral control measure assessed a decontextualized account of personal control over physical activity behavior. Consistent with previous research examining the differential effects of self-efficacy and perceived behavioral control on intentions and behaviors (Rodgers et al., 2008; Pertl et al., 2010), the results showed that self-efficacy had greater predictive utility for physical activity intentions, behavioral processes of change, and physical activity behavior, providing additional evidence that these social cognition constructs can be reliably distinguished from one another. Moreover, subjective norms provided a stronger predictive account of the behavioral processes of change than physical activity intentions. It is possible that greater endorsement of subjective norms leads individuals to enlist more support for physical activity engagement through the helping relationships process of change.

The findings of differential predictions for intentions and processes of change not only speak to the importance of examining intentions (as is common practice), but also incorporating belief-relevant strategies that enhance the probability of following through on one’s intentions. For example, individuals who value the stress relief benefits of physical activity could be encouraged to create plans to engage in physical activity immediately after time spent in stressful contexts (e.g., work, childcare, school). On the other hand, individuals who value the mental clarity and productivity that may come from physical activity behavior may be encouraged to plan physical activity into a morning routine to experience these benefits throughout the day. Furthermore, although intentions strongly predict behavior, they can also be susceptible to changes and fluctuations over time. Many individuals are unable to translate physical activity intentions into actual behavior (Rhodes et al., 2003; Rhodes and de Bruijn, 2013). The present study suggests that combining physical activity intentions with more concrete action steps (such as the behavioral processes of change) might be beneficial in bridging the intention-behavior gap (Rhodes and Dickau, 2012).

Aside from the direct paths identified in the Disposition-Belief-Motivation model, significant indirect effects were also found among the study variables. Both the activity facet and industriousness showed significant indirect effects to physical activity intentions, behavioral processes of change, and physical activity behavior, although the effects for industriousness were stronger than for the activity facet. This difference may be due to the propensity of industrious individuals to be deliberate and planful in their beliefs and motivations in relation to goal-related behaviors. As such, industrious individuals appear to be more likely to consider several forms of evaluation of a specific behavior, such as physical activity. This suggests that although not all individuals high on industriousness strive to achieve health-related (or physical-activity-related) goals, for some industrious individuals, maintaining health through physical activity is likely evaluated as an instrumental means to maintain and enhance everyday functioning to achieve other long-term goals. On the other hand, individuals who are naturally active and energetic may rely more on their affective evaluations of how physical activity makes them feel and are more confident in their ability to be active due to these activities already being familiar to them (Rhodes et al., 2005).

### Limitations and Implications

There are two primary limitations that should be noted in the interpretation of the results; the cross-sectional design and the use of self-reports for physical activity behavior. Future studies should incorporate objective measures of activity and fitness, such as accelerometer-based readings of physical activity and/or maximal oxygen consumption, as well as informant reports of trait facets. A possible secondary limitation is predictor-criterion contamination for the assessment of the trait activity facet and the assessment of engagement in physical activity. These two variables are not generally considered to be isomorphic. As a result, prior research examining trait activity and physical activity has not omitted select activity facet items in the course of conducting analyses including these two variables. Moreover, as the content of the two measures suggests, a person who is “always busy” or “always on the go” (positively scored trait activity items) might not necessarily be staying busy with leisure-time physical activity, while on the other hand, a person who likes a “leisurely lifestyle” and/or to “take it easy” (reverse-scored trait activity items) might perceive engagement in physical activity as part of a leisurely lifestyle. Furthermore, the instructions and available responses for the two variables are quite distinct, and the 0.13 correlation between trait activity and physical activity reported in the present work provides further confidence that two these variables measure unique aspects of dispositional tendencies and discrete behaviors that are neither interchangeable nor unduly conflated.

Despite the limitations, given the large representative sample and breadth of assessment, the data and methods were appropriate for addressing the research questions. The theoretical frameworks used in the present study befit the goal of testing structural models of trait facets, social cognitions, and physical activity behavior. Ultimately, both the Theory of Planned Behavior and Neo-Socioanalytic Theory attempt to describe how discrete behaviors are enacted (Ajzen, 1991; Roberts and Wood, 2006) – a necessary precedent for designing more complete predictive and intervention-based studies of health-related behaviors, such as physical activity. Although the following section provides suggestions for how the Disposition-Belief-Motivation model could be tested, it is important to note more work is needed to confirm the directionality of the variables in the model.

Many intervention paradigms have been developed using Theory of Planned Behavior constructs because of their predictive utility and intuitive appeal. The present research adds to the evidence supporting relationships from Theory of Planned Behavior constructs (most notably, intentions) to physical activity behavior. However, a noted limitation is that predictions from social cognition constructs alone may lack temporal stability (Ajzen, 2002, 2011). Although physical activity intentions strongly predicted physical activity behavior, they are nonetheless susceptible to change. The present research provides an elaborated account of physical activity intentions, underscoring the comparatively stable indirect influences of personality traits and the less stable, but direct influences of affective attitudes, outcome expectances, perceived behavioral control, and self-efficacy.

Perhaps more important than the various predictive paths described above, however, is the integration of core components from three social cognition theories with trait facets and physical activity behavior to produce a more comprehensive model. Although there have been calls for greater theoretical testing, modification, and integration in order to reduce the number of theories with overlapping constructs (Michie et al., 2005), this type of integration has not been undertaken in prior research. The present research adds to the evidence supporting the original Theory of Planned Behavior structure, as well as placing emphasis upon other social cognitions that warrant more consistent inclusion in future research. As a whole, the Disposition-Belief-Motivation model serves as a viable conceptual platform for new research to address recent calls for more instrumental, process-based accounts of trait-health relations (Hampson, 2012; Bogg and Roberts, 2013) The pathways identified in the Disposition-Belief-Motivation model should be examined using longitudinal and experimental designs in order to validate the directionality of the model variables and to investigate which constructs are most influential at different phases of physical activity initiation and maintenance. Research has shown that several influences, either in opposition to or in conjunction with others, can help individuals initiate or increase physical activity behavior. For example, research has shown that framing an exercise message through affective communication increases self-reported exercise levels more than through cognitive communication (Conner et al., 2011). A randomized controlled trial showed that the creation of written, detailed plans (i.e., implementation intentions) was able to increase exercise behavior (Andersson and Moss, 2011). Other studies have shown that combinations of influences can further enhance physical activity adoption, such as building self-efficacy in tandem with creating exercise plans and goals (Schwarzer et al., 2007; Renner et al., 2012). However, in spite of some of the advances in understanding influences on the initiation of and/or short-term increases in physical activity, the mechanisms through which physical activity can be maintained over the long term remain unknown.

Longitudinal tests of the Disposition-Belief-Motivation model of trait facets and social cognitions might help elucidate candidate intervention modalities for both physical activity initiation and maintenance. For example, among individuals high on the activity facet and low on physical activity, a pathway to increased physical activity engagement and maintenance might be implemented by emphasizing physical activity’s consistency with their general identity and active proclivities. Moreover, to the extent individuals can be trained to make accurate self-appraisals of levels of industriousness to aid in the realistic formulation of physical activity attitudes, expectancies, self-efficacy, behavioral intentions, and strategies to engage in physical activity, then utilizing a personality-informed approach for increasing physical activity might enable individuals to formulate physical activity goals that are more closely aligned with their dispositions, beliefs, and motivations – and importantly, are sustainable beyond the short term.

## Conflict of Interest Statement

The authors declare that the research was conducted in the absence of any commercial or financial relationships that could be construed as a potential conflict of interest.
