# Supplementary material for: Testing Theory of Planned Behavior and Neo-Socioanalytic Theory models of trait activity, industriousness, exercise social cognitions, exercise intentions, and physical activity in a representative U.S. sample
Source: Front Psychol. 2015 Aug 6;6:1114. doi: 10.3389/fpsyg.2015.01114 (PMC4526790; doi:10.3389/fpsyg.2015.01114)
Supplement: Supplementary file 1 [file Data_Sheet_1.DOCX]

*Variance-Covariance matrix of study variables.*

|  | Activity | Industriousness | Outcome Expectancies | Norms | Self-Efficacy | Affective Attitudes | Perceived Behavioral Control | Intention | Behavioral Processes of Change | Physical Activity (Mod/Stren) |
| --- | --- | --- | --- | --- | --- | --- | --- | --- | --- | --- |
| Activity | 0.28591 | 0.11812 | 0.03328 | 0.02345 | 0.09153 | 0.11985 | 0.05710 | 0.12463 | 0.07630 | 0.22672 |
| Industriousness | 0.11812 | 0.41189 | 0.10047 | 0.08105 | 0.15029 | 0.13398 | 0.17031 | 0.25766 | 0.13121 | 0.21726 |
| Outcome Expectancies | 0.03328 | 0.10047 | 0.32381 | 0.25153 | 0.22547 | 0.30079 | 0.23606 | 0.47548 | 0.28531 | 0.51706 |
| Norms | 0.02345 | 0.08105 | 0.25153 | 1.06971 | 0.28654 | 0.32283 | 0.31432 | 0.63281 | 0.40737 | 0.70537 |
| Self-Efficacy | 0.09153 | 0.15029 | 0.22547 | 0.28654 | 0.97782 | 0.45992 | 0.27149 | 0.94071 | 0.47016 | 1.03105 |
| Affective Attitudes | 0.11985 | 0.13398 | 0.30079 | 0.32283 | 0.45992 | 1.07392 | 0.34525 | 1.05892 | 0.52177 | 1.19869 |
| Perceived Behavioral Control | 0.05710 | 0.17031 | 0.23606 | 0.31432 | 0.27149 | 0.34525 | 0.83769 | 0.62988 | 0.29270 | 0.65157 |
| Intention | 0.12463 | 0.25766 | 0.47548 | 0.63281 | 0.94071 | 1.05892 | 0.62988 | 4.45031 | 1.09730 | 4.25402 |
| Behavioral Processes of Change | 0.07630 | 0.13121 | 0.28531 | 0.40737 | 0.47016 | 0.52177 | 0.29270 | 1.09730 | 0.71921 | 1.34293 |
| Physical Activity (Mod/Stren) | 0.22672 | 0.21726 | 0.51706 | 0.70537 | 1.03105 | 1.19869 | 0.65157 | 4.25402 | 1.34293 | 9.95967 |
